# Crystal structure of 5-benzyl-8-bromo-2-meth­yl-1,3-oxazolo[4,5-*c*][1,8]naphthyridin-4(5*H*)-one

**DOI:** 10.1107/S2056989017005023

**Published:** 2017-04-11

**Authors:** Johannes Vrijdag, An Van den Bogaert, Wim De Borggraeve, Luc Van Meervelt

**Affiliations:** a KU Leuven - University of Leuven, Department of Chemistry, Celestijnenlaan 200F - bus 2404, B-3001 Heverlee, Belgium

**Keywords:** crystal structure, benzodiazepine drugs, oxazolonaphthyridone, π–π stacking

## Abstract

The title compound, C_17_H_12_BrN_3_O_2_, was unexpectedly isolated during an attempt to synthesize pyridodiazepinediones and identified as an oxazolonaphthyridinone derivative. The almost planar oxazolonaphthyridinone ring (r.m.s. deviation = 0.016 Å) makes a dihedral angle of 61.6 (2)° with the phenyl ring. In the crystal, columns of mol­ecules stacked along the *a* axis are formed by π–π inter­actions between the six-membered rings of the oxazolonaphthyridone moieties [centroid-to-centroid distances = 3.494 (2)–3.906 (3) Å], which further inter­act through C—H⋯π contacts with the phenyl rings.

## Abstract 2

The structure of an oxazolonaphthyridinone derivative unexpectedly formed during the synthesis of pyridodiazepinediones is reported.

## Chemical context   

**Figure 1:**
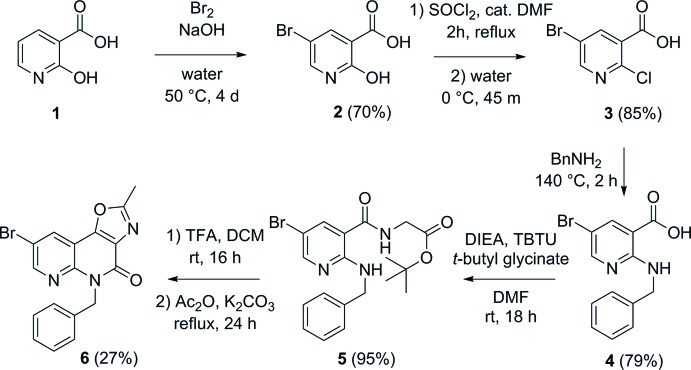
Synthesis of the title compound **6** as unexpectedly formed during the synthesis of pyridodiazepinediones.

While benzodiazepine drugs have been amongst the most prescribed medication globally since their discovery in the 1950s, the search for structurally related biologically active compounds is of major relevance to the pharmaceutical industry (Washton & Zweben, 2011 ▸). Previous work in our group dealing with the construction of pyridodiazepinediones (PZDs; Van den Bogaert *et al.*, 2010 ▸) led unexpectedly to the isolation of a tricyclic compound, which was later identified as oxazolonaphthyridinone (ONO) **6** (Fig. 1 ▸). Commercially available 2-hy­droxy­nicotinic acid **1** was converted to dihalonicotinic acid **3** *via* two sequential halogenation reactions (Van den Bogaert *et al.*, 2010 ▸; Gero *et al.*, 1989 ▸; Haché *et al.*, 2002 ▸), after which a benzyl­amine substituent was introduced yielding the aza-anthranilic acid derivative **4**. Next, ester compound **5** was prepared from inter­mediate **4** and *tert*-butyl glycinate using a standard coupling procedure. Finally, *tert*-butyl ester **5** was deprotected *in situ* and reacted with acetic anhydride in the presence of potassium carbonate, yielding tricyclic compound **6**. After exploration and optimization of the revealed cascade reaction towards the closely related oxazolo­quinolinone scaffold (Vrijdag *et al.*, 2013 ▸), we decided to turn our attention to the remarkable tricyclic product **6** isolated during the initial investigation. The ONO structural motif contained in compound **6** is brought into relation with both anti­bacterial (Ratcliffe *et al.*, 2015 ▸) and histamine 4 receptor antagonist (Ho *et al.*, 2013 ▸) activities. Hence, new synthetic routes towards ONOs are currently being developed in our laboratory (Vrijdag *et al.*, 2017 ▸). Here we present the mol­ecular and crystal structure of the title compound **6**.
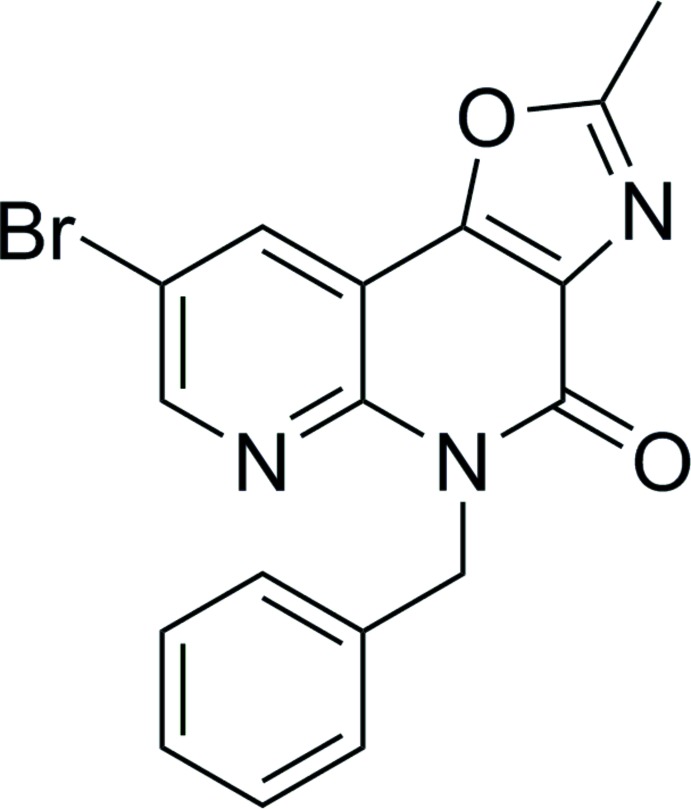


## Structural commentary   

**Figure 2:**
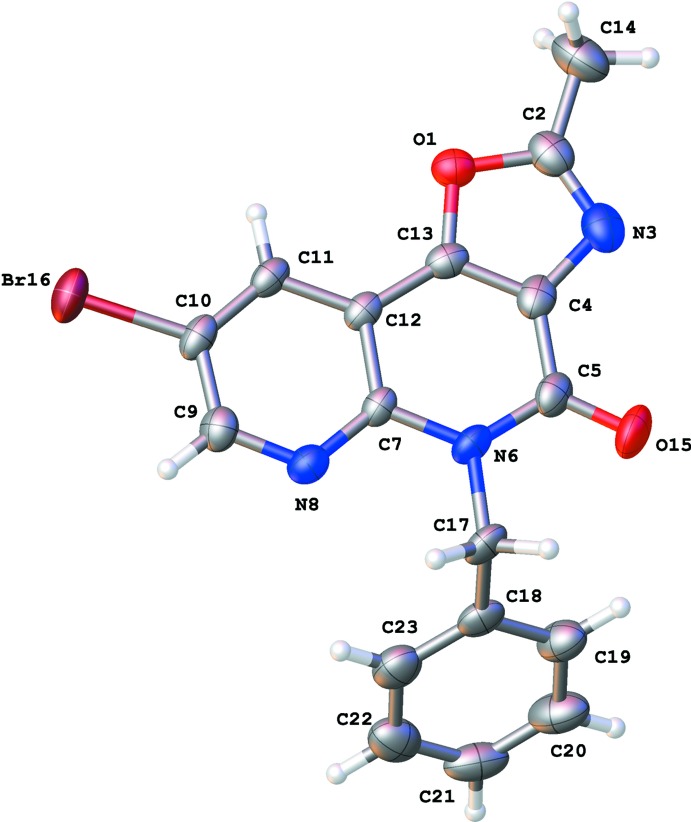
View of the asymmetric unit of the title compound **6**, showing the atom-labelling scheme. Displacement ellipsoids are drawn at the 50% probability level. H atoms are shown as small circles of arbitrary radii.

Crystals of **6** belong to the ortho­rhom­bic space group *Pna*2_1_ with one mol­ecule in the asymmetric unit (Fig. 2 ▸). The oxazolonaphthyridine ring is almost planar (r.m.s. deviation = 0.016 Å) with the substituents C14 [0.082 (6) Å], O15 [−0.023 (4) Å], Br16 [−0.012 (1) Å] and C17 [0.034 (5) Å] situated in the same plane (deviations from plane given in parenthesis). The dihedral angle between the mean planes through the oxazole and pyridine rings is 2.0 (2)°. The dihedral angle between the oxazolonaphthyridine ring system and the phenyl rings is 61.6 (2)°. Both H atoms of C17 are in close contact with the neighboring atoms N8 and O15 (H17*A*⋯N8 = 2.36 Å and H17*B*⋯O15 = 2.36 Å). No classical hydrogen bonds are observed.

## Supra­molecular features   

**Figure 3:**
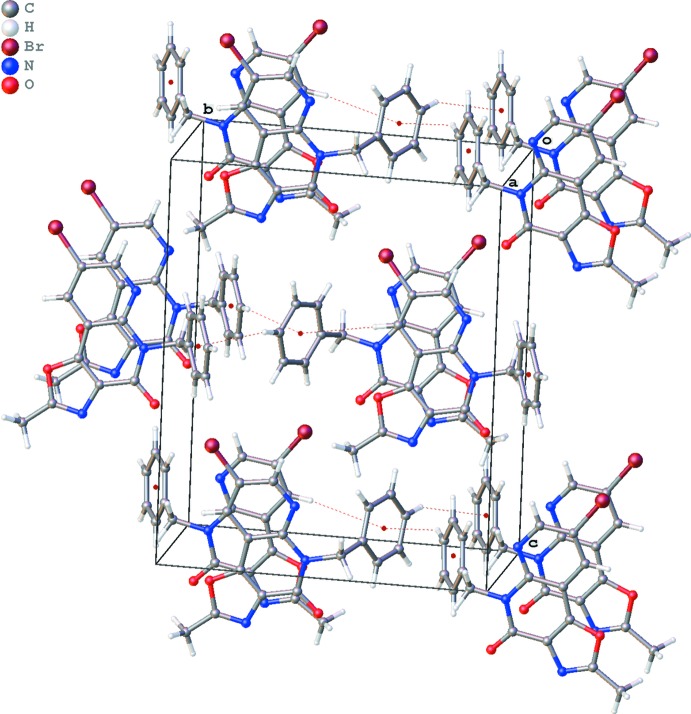
View of the crystal packing for the title compound **6**, showing C—H⋯π inter­actions (red dotted lines) between the C18–C23 phenyl rings.

**Figure 4:**
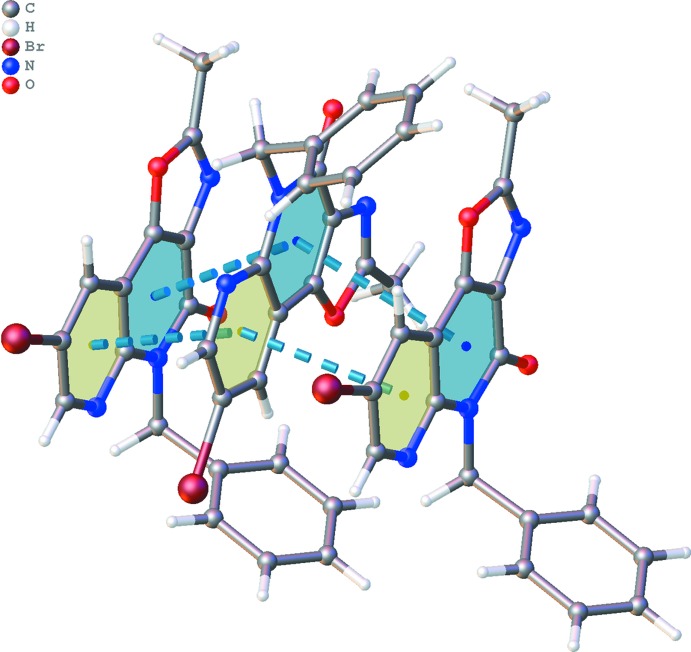
Part of the crystal packing of the title compound **6**, showing π–π inter­actions between the C7/N8/C9–C12 (blue) and C4/C5/N6/C7/C12/C13 (yellow) rings.

The crystal packing (Fig. 3 ▸) is characterized by π–π inter­actions between the six-membered rings of the oxazolonaphthyridone ring systems, resulting in columns of stacked mol­ecules along the *a* axis [Fig. 4 ▸; *Cg*1⋯*Cg*1^i^ = 3.494 (2) Å and *Cg*2⋯*Cg*2^i^ = 3.906 (3) Å; *Cg*1 and *Cg*2 are the centroids of the rings C7/N8/C9–C12 and C4/C5/N6/C7/C12/C13, respectively; symmetry code: (i) *x* + 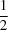, −*y* + 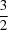, *z*]. Mol­ecules in neighboring columns show further C—H⋯π inter­actions between the C18–C23 phenyl rings (Fig. 3 ▸, Table 1 ▸). The closest contact of Br16 in the packing is with atom O15^ii^ [2.874 (4) Å; symmetry code: (ii) −*x* + 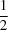, *y* − 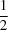, *z* − 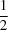].

## Database survey   

A search of the Cambridge Structural Database (CSD, Version 5.38, last update February 2017; Groom *et al.*, 2016 ▸) for a [1,3]oxazolo[4,5-*c*]-1,8-naphthyridin-4(5*H*)-one ring skeleton gave no hits. The closest ring skeleton is found in 2,5-dimeth­yl[1,3]oxazolo[4,5-c]quinolin-4(5*H*)-one (refcode HOJTUW; Latypov *et al.*, 2008 ▸), which contains a quinolinone ring system instead of a naphthyridinone ring system. The oxazolo­quinoline ring is almost planar (r.m.s. deviation = 0.015 Å) with a dihedral angle between the oxazole and phenyl rings of 1.90 (13)°.

## Synthesis and crystallization   

**Synthesis of 5-bromo-2-hy­droxy­nicotinic acid (2), 5-bromo-2-chloro­nicotinic acid (3), and 2-(benzyl­amino)-5-bromo­nicotinic acid (4):**

Substituted nicotinic acids **2–4** were synthesized following the protocols of Van den Bogaert *et al.* (2010 ▸). Analytical data matches literature data.

**Synthesis of** ***tert*** **-but­yl** ***N*** **-{[2-(benzyl­amino)-5-bromo­pyridin-3-yl]carbon­yl}glycinate (5):**

2-(Benzyl­amino)-5-bromo­nicotinic acid **4** (50 mg, 0.16 mmol) was dissolved in di­methyl­formamide under an Ar atmosphere, and di-iso­propyl­ethyl­amine (27 µl, 0.16 mmol) and benzotriazolyl tetra­methyl­uronium fluoro­borate (TBTU, 57 mg, 0.18 mmol) were subsequently added to the mixture. The reaction was stirred at room temperature for 15 m, and *t*-butyl glycinate (24 µl, 0.18 mmol) was added. The reaction was continued at room temperature for 18 h, after which the mixture was concentrated under reduced pressure. The residue was purified using silica gel chromatography (hepta­ne/ethyl acetate, 8:2 *v*/*v*) to yield compound **5** (64 mg, yield 95%).

IR (Perkin–Elmer 1720 FTIR, KBr, cm^−1^): ν = 1705 (*s*, CO ester), 1648 (*s*, CO amide). ^1^H NMR [Bruker 400 Avance, 400 MHz, CDCl_3_, δ (ppm), *J* (Hz)]: 8.42 (*t*, 1H, *J* = 5, CH), 8.21 (*d*, 1H, *J* = 2, CH), 7.76 (*d*, 1H, *J* = 2, CH), 7.34–7.22 (*m*, 5H, CH), 6.84 (*t*, 1H, *J* = 5, CH), 4.65 (*d*, 2H, *J* = 6, CH_2_), 4.02 (*d*, 2H, *J* = 5, CH_2_), 1.49 (*s*, 9H, CH_3_). ^13^C NMR [Bruker 400 Avance, 101 MHz, CDCl_3_, δ (ppm)]: 169.4, 167.1, 156.3, 152.6, 139.3, 137.6, 128.6, 127.6, 127.1, 110.6, 104.4, 82.9, 45.0, 42.3, 28.1.

**Synthesis of 5-benzyl-8-bromo-2-meth­yl[1,3]oxazolo[4,5-** ***c*** **]-1,8-naphthyridin-4(5** ***H*** **)-one (6):**

A mixture of *tert*-butyl *N*-{[2-(benzyl­amino)-5-bromo­pyridin-3-yl]carbon­yl}glycinate **5** (50 mg, 0.12 mmol) and di­chloro­methane (2.25 mL) was cooled to 273 K, after which tri­fluoro­acetic acid (0.75 mL) was added. The reaction was continued at room temperature for 16 h, concentrated under reduced pressure, and dried under high vacuum. The obtained crude acid was combined with K_2_CO_3_ (38 mg, 0.28 mmol) and acetic anhydride (0.5 mL) under an Ar atmosphere and the mixture was stirred at room temperature for 30 m. Subsequently the reaction was heated to reflux for 24 h, after which the mixture was concentrated under reduced pressure. The residue was purified using silica gel chromatography (di­chloro­methane/methanol, 99:1 *v*/*v*) to yield the title compound (12 mg, yield 27%). Light-brown prismatic crystals were grown by diffusion of pentane in a chloro­form solution of the title compound.

IR (Perkin–Elmer 1720 FTIR, NaCl, cm^−1^): ν = 1683 (*s*, CO amide). ^1^H NMR [Bruker 400 Avance, 400 MHz, CDCl_3_, δ (ppm), *J* (Hz)]: 8.65 (*d*, 1H, *J* = 2, CH), 8.27 (*d*, 1H, *J* = 2, CH), 7.48 (*dd*, 2H, *J* = 7, 1, CH), 7.26–7.21 (*m*, 3H, CH), 5.80 (*s*, 2H, CH_2_), 2.71 (*s*, 3H, CH_3_). ^13^C NMR [Bruker 400 Avance, 101 MHz, CDCl_3_, δ (ppm)]: 164.6, 157.3, 150.2, 149.9, 146.6, 137.5, 131.5, 131.0, 128.9, 128.4, 127.5, 113.9, 108.5, 44.8, 14.5.

## Refinement   

Crystal data, data collection and structure refinement details are summarized in Table 2 ▸. All H atoms were placed in calculated positions with C—H = 0.95 Å for aromatic, C—H = 0.98 Å for CH_3_ or C—H = 0.99 Å for CH_2_ H atoms, and included in the refinement in a riding model with *U*_iso_(H) = 1.2 or 1.5*U*_eq_(C).

## Supplementary Material

Crystal structure: contains datablock(s) I. DOI: 10.1107/S2056989017005023/rz5210sup1.cif

Structure factors: contains datablock(s) I. DOI: 10.1107/S2056989017005023/rz5210Isup2.hkl

Supporting information file. DOI: 10.1107/S2056989017005023/rz5210Isup3.cml

CCDC reference: 1541539

Additional supporting information:  crystallographic information; 3D view; checkCIF report

## Figures and Tables

**Table 1:** Hydrogen-bond geometry (Å, °) *Cg*3 is the centroid of the C18–C23 ring.

*D*—H⋯*A*	*D*—H	H⋯*A*	*D*⋯*A*	*D*—H⋯*A*
C21—H21⋯*Cg*3^i^	0.95	2.82	3.604 (6)	141
C11—H11⋯*Cg*3^ii^	0.95	3.31	4.239 (6)	167

Symmetry codes: (i) 
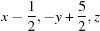
; (ii) 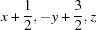.

**Table 2:** Experimental details

Crystal data	
Chemical formula	C_17_H_12_BrN_3_O_2_
*M*_r_	370.21
Crystal system, space group	Orthorhombic, *P* *n* *a*2_1_
Temperature (K)	200
*a*, *b*, *c* (Å)	6.7150 (13), 13.504 (3), 16.757 (3)
*V* (Å^3^)	1519.5 (5)
*Z*	4
Radiation type	Mo *K*α
μ (mm^−1^)	2.72
Crystal size (mm)	0.3 × 0.3 × 0.2

Data collection	
Diffractometer	Enraf–Nonius CAD-4
Absorption correction	ψ scan (North *et al.*, 1968 ▸)
*T*_min_, *T*_max_	0.522, 0.578
No. of measured, independent and observed [*I* > 2σ(*I*)] reflections	1429, 1429, 1279
*R*_int_	0.049
(sin θ/λ)_max_ (Å^−1^)	0.601

Refinement	
*R*[*F* ^2^ > 2σ(*F* ^2^)], *wR*(*F* ^2^), *S*	0.027, 0.069, 1.16
No. of reflections	1429
No. of parameters	209
No. of restraints	1
H-atom treatment	H-atom parameters constrained
Δρ_max_, Δρ_min_ (e Å^−3^)	0.34, −0.27
Absolute structure	No quotients, so Flack parameter determined by classical intensity fit
Absolute structure parameter	0.000 (12)

Computer programs: *CAD-4 EXPRESS* (Enraf–Nonius, 1989 ▸), *DREAR* (Blessing, 1987 ▸), *SHELXS* (Sheldrick, 2008 ▸), *SHELXL2014/7* (Sheldrick, 2015 ▸) and *OLEX2* (Dolomanov *et al.*, 2009 ▸).

## supplementary crystallographic information

### Crystal data

**Table:**

C_17_H_12_BrN_3_O_2_	*D*_x_ = 1.618 Mg m^−^^3^
*M**_r_* = 370.21	Mo *K*α radiation, λ = 0.71073 Å
Orthorhombic, *P**n**a*2_1_	Cell parameters from 25 reflections
*a* = 6.7150 (13) Å	θ = 1.9–25.3°
*b* = 13.504 (3) Å	µ = 2.72 mm^−^^1^
*c* = 16.757 (3) Å	*T* = 200 K
*V* = 1519.5 (5) Å^3^	Prism, light brown
*Z* = 4	0.3 × 0.3 × 0.2 mm
*F*(000) = 744	

### Data collection

**Table:**

Enraf–Nonius CAD-4 diffractometer	1279 reflections with *I* > 2σ(*I*)
Radiation source: fine-focus sealed tube	*R*_int_ = 0.049
Graphite monochromator	θ_max_ = 25.3°, θ_min_ = 1.9°
ω/2θ scans	*h* = 0→8
Absorption correction: ψ scan (North *et al.*, 1968)	*k* = 0→16
*T*_min_ = 0.522, *T*_max_ = 0.578	*l* = 0→20
1429 measured reflections	3 standard reflections every 97 reflections
1429 independent reflections	intensity decay: 0.5%

### Refinement

**Table:**

Refinement on *F*^2^	Hydrogen site location: inferred from neighbouring sites
Least-squares matrix: full	H-atom parameters constrained
*R*[*F*^2^ > 2σ(*F*^2^)] = 0.027	*w* = 1/[σ^2^(*F*_o_^2^) + (0.0221*P*)^2^ + 0.5935*P*] where *P* = (*F*_o_^2^ + 2*F*_c_^2^)/3
*wR*(*F*^2^) = 0.069	(Δ/σ)_max_ < 0.001
*S* = 1.16	Δρ_max_ = 0.34 e Å^−^^3^
1429 reflections	Δρ_min_ = −0.27 e Å^−^^3^
209 parameters	Absolute structure: No quotients, so Flack parameter determined by classical intensity fit
1 restraint	Absolute structure parameter: 0.000 (12)
Primary atom site location: structure-invariant direct methods	

### Special details

**Table:**

Geometry. All esds (except the esd in the dihedral angle between two l.s. planes) are estimated using the full covariance matrix. The cell esds are taken into account individually in the estimation of esds in distances, angles and torsion angles; correlations between esds in cell parameters are only used when they are defined by crystal symmetry. An approximate (isotropic) treatment of cell esds is used for estimating esds involving l.s. planes.

### Fractional atomic coordinates and isotropic or equivalent isotropic displacement parameters (Å^2^)

**Table:**

	*x*	*y*	*z*	*U*_iso_*/*U*_eq_	
O1	0.1853 (5)	0.6350 (2)	1.0622 (2)	0.0353 (7)	
C2	0.1680 (7)	0.6538 (4)	1.1434 (3)	0.0408 (11)	
N3	0.1585 (6)	0.7463 (3)	1.1614 (2)	0.0430 (10)	
C4	0.1733 (6)	0.7940 (4)	1.0879 (3)	0.0344 (10)	
C5	0.1745 (8)	0.8991 (4)	1.0713 (3)	0.0347 (11)	
N6	0.1944 (5)	0.9220 (3)	0.9912 (2)	0.0311 (8)	
C7	0.2122 (7)	0.8525 (3)	0.9299 (3)	0.0261 (10)	
N8	0.2338 (5)	0.8876 (3)	0.8560 (3)	0.0324 (8)	
C9	0.2503 (6)	0.8216 (4)	0.7968 (3)	0.0352 (10)	
H9	0.2659	0.8454	0.7438	0.042*	
C10	0.2456 (6)	0.7205 (3)	0.8090 (3)	0.0327 (10)	
C11	0.2253 (6)	0.6834 (3)	0.8840 (3)	0.0299 (9)	
H11	0.2232	0.6140	0.8929	0.036*	
C12	0.2078 (6)	0.7493 (3)	0.9470 (2)	0.0269 (9)	
C13	0.1890 (6)	0.7265 (3)	1.0287 (3)	0.0299 (9)	
C14	0.1651 (9)	0.5668 (5)	1.1959 (4)	0.0590 (16)	
H14A	0.1328	0.5876	1.2504	0.089*	
H14B	0.2962	0.5350	1.1953	0.089*	
H14C	0.0643	0.5199	1.1770	0.089*	
O15	0.1582 (6)	0.9635 (3)	1.1226 (2)	0.0501 (9)	
Br16	0.26869 (6)	0.63431 (3)	0.72061 (4)	0.04509 (16)	
C17	0.2032 (7)	1.0293 (3)	0.9709 (3)	0.0359 (10)	
H17A	0.2912	1.0383	0.9241	0.043*	
H17B	0.2634	1.0656	1.0163	0.043*	
C18	0.0019 (6)	1.0740 (3)	0.9526 (3)	0.0360 (10)	
C19	−0.1165 (8)	1.1102 (4)	1.0132 (4)	0.0456 (13)	
H19	−0.0752	1.1033	1.0671	0.055*	
C20	−0.2946 (10)	1.1562 (5)	0.9957 (5)	0.0567 (18)	
H20	−0.3748	1.1810	1.0379	0.068*	
C21	−0.3576 (8)	1.1669 (4)	0.9187 (4)	0.0534 (15)	
H21	−0.4804	1.1990	0.9075	0.064*	
C22	−0.2419 (8)	1.1307 (4)	0.8573 (4)	0.0514 (16)	
H22	−0.2840	1.1380	0.8035	0.062*	
C23	−0.0632 (8)	1.0834 (4)	0.8746 (3)	0.0476 (13)	
H23	0.0152	1.0572	0.8324	0.057*	

### Atomic displacement parameters (Å^2^)

**Table:**

	*U*^11^	*U*^22^	*U*^33^	*U*^12^	*U*^13^	*U*^23^
O1	0.0308 (15)	0.0340 (17)	0.0412 (18)	0.0005 (12)	−0.0015 (14)	0.0023 (14)
C2	0.029 (2)	0.053 (3)	0.040 (3)	0.003 (2)	−0.001 (2)	0.006 (2)
N3	0.031 (2)	0.060 (3)	0.037 (2)	0.0057 (18)	−0.0035 (18)	−0.001 (2)
C4	0.022 (2)	0.043 (3)	0.038 (3)	0.006 (2)	−0.0047 (19)	−0.008 (2)
C5	0.028 (3)	0.039 (3)	0.037 (3)	0.001 (2)	−0.008 (2)	−0.012 (2)
N6	0.0261 (17)	0.0250 (18)	0.042 (2)	−0.0009 (15)	0.0004 (16)	−0.0089 (16)
C7	0.018 (2)	0.028 (2)	0.033 (2)	−0.0013 (18)	0.0012 (19)	−0.0084 (19)
N8	0.0262 (17)	0.030 (2)	0.041 (2)	0.0006 (14)	0.0026 (16)	−0.0028 (17)
C9	0.028 (2)	0.042 (3)	0.035 (2)	0.0009 (19)	0.0039 (19)	−0.006 (2)
C10	0.022 (2)	0.034 (2)	0.042 (2)	−0.0003 (17)	0.0011 (18)	−0.0154 (19)
C11	0.022 (2)	0.027 (2)	0.041 (2)	0.0004 (18)	−0.0027 (18)	−0.0099 (19)
C12	0.0164 (19)	0.027 (2)	0.037 (2)	−0.0007 (15)	−0.0006 (17)	−0.0069 (18)
C13	0.0204 (19)	0.027 (2)	0.043 (3)	0.0006 (16)	−0.0016 (18)	−0.0008 (18)
C14	0.053 (3)	0.072 (4)	0.053 (3)	0.006 (3)	−0.003 (2)	0.022 (3)
O15	0.057 (2)	0.049 (2)	0.044 (2)	0.0047 (18)	−0.0070 (18)	−0.0256 (17)
Br16	0.0427 (2)	0.0502 (3)	0.0424 (2)	−0.0015 (2)	0.0038 (4)	−0.0214 (3)
C17	0.036 (2)	0.023 (2)	0.049 (3)	−0.0021 (17)	0.002 (2)	−0.011 (2)
C18	0.035 (2)	0.020 (2)	0.052 (3)	−0.0044 (17)	0.002 (2)	−0.002 (2)
C19	0.045 (3)	0.038 (3)	0.054 (3)	0.007 (2)	0.011 (3)	0.002 (2)
C20	0.049 (4)	0.043 (3)	0.078 (5)	0.013 (3)	0.014 (4)	0.008 (3)
C21	0.037 (3)	0.032 (3)	0.092 (5)	0.001 (2)	−0.009 (3)	0.007 (3)
C22	0.056 (4)	0.040 (3)	0.058 (4)	−0.010 (3)	−0.011 (3)	0.006 (2)
C23	0.053 (3)	0.033 (3)	0.056 (3)	−0.003 (2)	0.003 (3)	−0.003 (2)

### Geometric parameters (Å, º)

**Table:**

O1—C2	1.389 (6)	C11—C12	1.386 (6)
O1—C13	1.358 (5)	C12—C13	1.408 (6)
C2—N3	1.287 (6)	C14—H14A	0.9800
C2—C14	1.467 (7)	C14—H14B	0.9800
N3—C4	1.393 (6)	C14—H14C	0.9800
C4—C5	1.446 (7)	C17—H17A	0.9900
C4—C13	1.352 (6)	C17—H17B	0.9900
C5—N6	1.384 (7)	C17—C18	1.512 (6)
C5—O15	1.229 (6)	C18—C19	1.379 (7)
N6—C7	1.396 (6)	C18—C23	1.383 (7)
N6—C17	1.489 (6)	C19—H19	0.9500
C7—N8	1.335 (7)	C19—C20	1.380 (9)
C7—C12	1.423 (6)	C20—H20	0.9500
N8—C9	1.338 (6)	C20—C21	1.365 (10)
C9—H9	0.9500	C21—H21	0.9500
C9—C10	1.381 (7)	C21—C22	1.379 (9)
C10—C11	1.359 (7)	C22—H22	0.9500
C10—Br16	1.890 (4)	C22—C23	1.390 (7)
C11—H11	0.9500	C23—H23	0.9500
			
C13—O1—C2	103.9 (4)	C4—C13—C12	125.0 (4)
O1—C2—C14	116.2 (4)	C2—C14—H14A	109.5
N3—C2—O1	114.3 (4)	C2—C14—H14B	109.5
N3—C2—C14	129.5 (5)	C2—C14—H14C	109.5
C2—N3—C4	103.8 (4)	H14A—C14—H14B	109.5
N3—C4—C5	128.6 (4)	H14A—C14—H14C	109.5
C13—C4—N3	110.1 (4)	H14B—C14—H14C	109.5
C13—C4—C5	121.3 (4)	N6—C17—H17A	108.9
N6—C5—C4	114.0 (4)	N6—C17—H17B	108.9
O15—C5—C4	124.1 (5)	N6—C17—C18	113.6 (3)
O15—C5—N6	121.9 (5)	H17A—C17—H17B	107.7
C5—N6—C7	124.8 (4)	C18—C17—H17A	108.9
C5—N6—C17	116.3 (4)	C18—C17—H17B	108.9
C7—N6—C17	118.9 (4)	C19—C18—C17	120.4 (5)
N6—C7—C12	120.6 (4)	C19—C18—C23	118.8 (5)
N8—C7—N6	116.9 (4)	C23—C18—C17	120.8 (5)
N8—C7—C12	122.5 (4)	C18—C19—H19	119.9
C7—N8—C9	117.4 (4)	C18—C19—C20	120.2 (6)
N8—C9—H9	118.4	C20—C19—H19	119.9
N8—C9—C10	123.1 (5)	C19—C20—H20	119.4
C10—C9—H9	118.4	C21—C20—C19	121.1 (6)
C9—C10—Br16	119.4 (4)	C21—C20—H20	119.4
C11—C10—C9	120.3 (4)	C20—C21—H21	120.2
C11—C10—Br16	120.4 (3)	C20—C21—C22	119.5 (6)
C10—C11—H11	120.8	C22—C21—H21	120.2
C10—C11—C12	118.5 (4)	C21—C22—H22	120.2
C12—C11—H11	120.8	C21—C22—C23	119.5 (6)
C11—C12—C7	118.2 (4)	C23—C22—H22	120.2
C11—C12—C13	127.5 (4)	C18—C23—C22	120.8 (6)
C13—C12—C7	114.3 (4)	C18—C23—H23	119.6
O1—C13—C12	127.1 (4)	C22—C23—H23	119.6
C4—C13—O1	107.9 (4)		
			
O1—C2—N3—C4	−0.9 (6)	N8—C9—C10—C11	−0.6 (7)
C2—O1—C13—C4	−0.3 (4)	N8—C9—C10—Br16	179.6 (3)
C2—O1—C13—C12	179.0 (4)	C9—C10—C11—C12	0.6 (6)
C2—N3—C4—C5	−179.0 (5)	C10—C11—C12—C7	−0.1 (6)
C2—N3—C4—C13	0.7 (5)	C10—C11—C12—C13	−178.8 (4)
N3—C4—C5—N6	179.1 (4)	C11—C12—C13—O1	0.4 (7)
N3—C4—C5—O15	−1.4 (8)	C11—C12—C13—C4	179.7 (4)
N3—C4—C13—O1	−0.2 (5)	C12—C7—N8—C9	0.5 (6)
N3—C4—C13—C12	−179.6 (4)	C13—O1—C2—N3	0.8 (5)
C4—C5—N6—C7	0.0 (7)	C13—O1—C2—C14	−178.6 (4)
C4—C5—N6—C17	−178.1 (4)	C13—C4—C5—N6	−0.5 (6)
C5—C4—C13—O1	179.5 (4)	C13—C4—C5—O15	179.0 (5)
C5—C4—C13—C12	0.1 (7)	C14—C2—N3—C4	178.4 (5)
C5—N6—C7—N8	−178.8 (4)	O15—C5—N6—C7	−179.5 (5)
C5—N6—C7—C12	0.9 (7)	O15—C5—N6—C17	2.4 (7)
C5—N6—C17—C18	−91.6 (5)	Br16—C10—C11—C12	−179.7 (3)
N6—C7—N8—C9	−179.8 (4)	C17—N6—C7—N8	−0.8 (6)
N6—C7—C12—C11	179.8 (4)	C17—N6—C7—C12	179.0 (4)
N6—C7—C12—C13	−1.3 (6)	C17—C18—C19—C20	176.1 (5)
N6—C17—C18—C19	86.4 (5)	C17—C18—C23—C22	−175.5 (4)
N6—C17—C18—C23	−96.4 (5)	C18—C19—C20—C21	0.1 (9)
C7—N6—C17—C18	90.2 (5)	C19—C18—C23—C22	1.7 (7)
C7—N8—C9—C10	0.1 (6)	C19—C20—C21—C22	0.3 (10)
C7—C12—C13—O1	−178.4 (4)	C20—C21—C22—C23	0.4 (8)
C7—C12—C13—C4	0.9 (6)	C21—C22—C23—C18	−1.4 (8)
N8—C7—C12—C11	−0.5 (6)	C23—C18—C19—C20	−1.1 (7)
N8—C7—C12—C13	178.5 (4)		

### Hydrogen-bond geometry (Å, º)

Cg3 is the centroid of the C18–C23 ring.

**Table:**

*D*—H···*A*	*D*—H	H···*A*	*D*···*A*	*D*—H···*A*
C21—H21···*Cg*3^i^	0.95	2.82	3.604 (6)	141
C11—H11···*Cg*3^ii^	0.95	3.31	4.239 (6)	167

Symmetry codes: (i) *x*−1/2, −*y*+5/2, *z*; (ii) *x*+1/2, −*y*+3/2, *z*.
